# Effect of Polyaniline on Sulfur/Sepiolite Composite Cathode for Lithium-Sulfur Batteries

**DOI:** 10.3390/polym12040755

**Published:** 2020-03-31

**Authors:** Kalaiselvi Chelladurai, Priyanka Venkatachalam, Subadevi Rengapillai, Wei-Ren Liu, Chia-Hung Huang, Sivakumar Marimuthu

**Affiliations:** 1 #120, Energy Materials Lab, Department of Physics, Science Block, Alagappa University, Karaikudi 630 003, Tamil Nadu, India; kashikoushi@gmail.com (K.C.); emlphysicspri@yahoo.com (P.V.); 2 Department of Chemical Engineering, R&D Center for Membrane Technology, Research Center for Circular Economy, Chung-Yuan Christian University, Chung-Li 32023, Taiwan; wrliu@cycu.edu.tw; 3 Metal Industries Research and Development Centre, Kaohsiung 81160, Taiwan; chiahung@mail.mirdc.org.tw

**Keywords:** sepiolite, polyaniline, XRD, SEM, electrochemical studies

## Abstract

Composite materials with a stable network structure consisting of natural sepiolite (Sp) powders (both sieved sepiolite and post-treated sepiolite), sulfur(S), and conductive polymer Polyaniline (PAni) have been successfully synthesized using a simple heat treatment. The morphology of composites illustrates that the sepiolite is composed of many needle-like fibrous clusters. The initial discharge capacity of the post-treated sepiolite/sulfur/PAni composite is about 1230 mA h g^−1^ at 0.1 C, and it remains at 826 mA h g^−1^ even after 40 cycles with the corresponding coulombic efficiency above 97%. Such performance is attributed to the specific porous structure, outstanding adsorption characteristics, and excellent ion exchange capability of sepiolite, as well as the excellent conductivity of PAni. In addition, the PAni coating has a pinning effect on sulfur, which influences the consumption of the active mass and enhances the cycling constancy and the coulombic efficiency of the composite material at elevated current rates.

## 1. Introduction

Rechargeable Lithium-ion batteries (LIBs) are one of the remarkable power sources for electric vehicles and portable electronic devices. The increasing demands of advanced electronic devices and electric vehicles, the development of high energy density, low cost, and long cycling performance batteries are of great importance. Among the rechargeable lithium battery family, the Lithium-sulfur (Li–S) battery offers a high theoretical specific capacity of 1675 mA h g^−1^ and energy density of 2600 Wh kg^−1^ [1,2,3,4]. Besides the high theoretical capacity, sulfur has the advantages of abundance, non-toxicity, environmental friendliness, cost worthiness, and wide operating temperature range when used as the positive electrode [5,6]. However, there are several serious of problems in realizing the practical applications of the Li–S battery. First, the poor conductivity of sulfur and its discharge products leads to low utilization of the active material. Second, the huge volume expansion active material during lithiation/delithiation, which causes the structure of cathode; Third, the shuttle effect polysulfides, resulting in a rapid capacity fading and low columbic efficiency [7,8,9,10].

To confront the fact that sulfur containing organic compounds are highly electrically and ionically insulating and to enable a reversible electrochemical reaction at high current rates, carbon materials have been implemented [11]. The use of structured carbons with designed porosity and a high surface area for sulfur storage aids the sulfur in maintaining intimate contact with an electrically conductive additive [12]. They allow to encapsulate the sulfur particles within the cathode and to mitigate the lithium polysulfide (LiPS) dissolution as well as buffer the huge volume change of the active material during lithiation and delithiation process [13]. 

Sepiolite is a hydrated magnesium silicate clay mineral with layered chain structure and it portrays fibrous morphology. It could be an excellent matrix and absorbing material for Li–S batteries due to its large pore volume and ion transmission channel. Also, it enables good conversion efficiency of active material due to the strong adsorption of sepiolite and polysulfides. However, the sepiolite has poor electrical conductivity and its impurity leads to low coulombic efficiency [14,15,16].

On the other hand, in order to surmount the chronic technical issues impeding the Li–S technology from practical applications, the sulfur-conductive polymer composites started with the application of PAN. Since then, as an alternative to carbon coating, sulfur has also been embedded with various conductive polymers during the past decade [17,18,19,20]. The properties of conducting polymers depend strongly on the doping level, protonation level, ion size of dopant, and water content [21]. Zhou et al. explained S-PAni core–shell composites using chemical oxidative polymerisation method on the surface of sulfur nanospheres [22]. PAni enhances the conductivity while alleviating the diffusion of LiPSs [23,24]. A more efficient consumption of PAni as conductive medium to obstruct the LiPS suspension was initiated by Xiao et al. Moreover, PAni has a massive electrical conductivity, elevated chemical stability and simply synthesis that it can be able to manage oxidation state and degree of protonation [25]. The strong chemical binding nature of PAni is a key factor for to immobilize the polysulfide and easily transport the electron for enhancing the electrochemical performance [26].

In this work, S/PTSp/PAni composite material was prepared via simple heat treatment. The polymer molecular scaffold provides strong physical and chemical internment to the elemental sulfur and the resident polysulfide. In addition, the polymer matrix, clay mineral sepiolite, and nano structured sulfur allows for the reversible deposition of transitional polysulfide species during discharge, and their ensuing conversion during recharge within the polymer matrix, as well as an investigation of its physical and electrochemical properties as a cathode for lithium rechargeable batteries.

## 2. Experimental

### 2.1. Sepiolite Acid Treatment

Sieved 200-mesh sepiolite powder, Hydrochloric acid (HCl), de-ionized water and Polyaniline polymer was purchased from Sigma Aldrich (St. Louis, MI, USA). Acid treatment was passed out using 8 mole ratio of Hydrochloric acid was added with deionized water in a beaker without disconcerting at surrounding temperature for 24 h. The clay minerals were filtered and washed with de-ionized water for frequent times, and then desiccated at 40 °C in a vacuum oven at 10^−3^ Torr for 12 h. 

### 2.2. Thermal Activation Method

The post-treated sieved sepiolite powders were composed according to the above treatment. In this process, 8 mole ratios of post-treated sepiolite, sublimed sulfur, and PAni (7:2:1) were passed through fine grinding for 1 h and then heated at 155 °C for 20 h in a muffle furnace. The collected sample was milled and dried to the get final product.

### 2.3. Characterization

The X-ray diffraction patterns and functional groups of calcined powder samples were examined by using X-ray diffraction (PAN alytical XPERT-PRO with Cu-Kα radiation, Malvem panalytical, Leyweg, EA Almelo, The Netherlands) and FTIR spectrometer recording IR spectra in the range of 4000–500 cm^−1^ using (Thermo Nicolet 380 Instrument Cooperation and KBr pellets, Woodland, CA, USA). The morphology of the powders was inspected by scanning electron microscope (EV018 (CARL ZEISS) Jena, Germany) and transmission electron microscope (TEM, JEOL, Musashino, Akishima-Shi, Tokyo, Japan) with energy dispersive X-ray spectroscopy (EDS). Raman spectroscopy (Renishaw inVia, excited by a 514 nm Ar-ion laser with a laser spot size of ~1 μm^2^, Japan) was used to characterize the electron shift. X-ray photoelectron spectroscopy (XPS) data were obtained employing a Phi 5300 X-ray Photoelectron Spectrometer (THERMO SCIENTIFIC, Waltham, MA, USA) with Mg K-alpha X-rays at an accelerating voltage of 15.0 kV (*h*_ν_ = 1253.6 eV) in a chamber maintained at 10^−9^ Torr.

### 2.4. Electrochemical Performance

Positive electrodes comprised of 70 wt % S/PTSp/PAni composite, 20 wt % acetylene black and 10 wt % polyvinylidene fluoride binders were evenly mixed in an *N*-methyl-2-pyrrolidone (NMP) solvent under incessant magnetic stirring for 3 h, and then the slurry was homogeneously coated on an aluminum foil. The electrodes were dried under vacuum at 60 °C for 12 h. CR2032 type coin cells were assembled in an argon-filled glove box, where lithium metal was used as a counter electrode and polypropylene membrane was used as the separator. The electrolyte was 1 M lithium bis (tri-fluoro methane sulfonyl) imide (LiTFSI) dissolved in a 1:1 volume ratio mixture of 1, 3-dioxolane and dimethoxy ethane. Electrochemical charge–discharge and cyclic performances were calculated at room temperature using a BTS-5V3A battery test system (Neware, Shenzhen, China), with the potential range from 3.0 to 1.5 V (vs. Li/Li^+^). 

## 3. Results and Discussion

### 3.1. XRD Analysis

**Figure 1:**
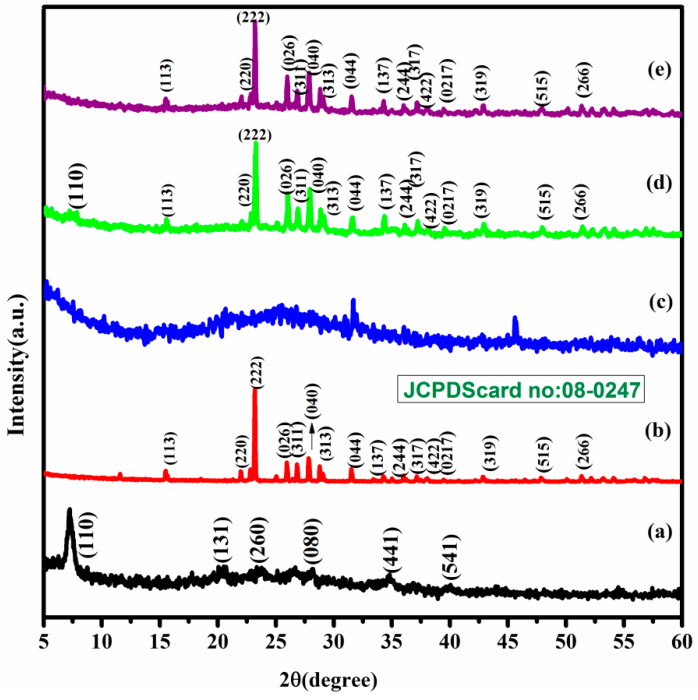
Patterns of (**a**) sieved sepiolite (SvSp), (**b**) pure sulfur (PS), (**c**) Polyaniline (PAni), (**d**) sulfur/sieved sepiolite/PAni (S/SvSp/PAni), (**e**) sulfur/post-treated sepiolite/PAni(S/PTSp/PAni).

The XRD patterns of SvSp, pure sulfur, PAni and composite materials such as S/PTSp/PAni and S/SvSp/PAni are shown in Figure 1. From Figure 1a it was observed that sample exhibits a strong diffraction peaks appeared at 2*θ* = 7.45° which corresponds to the (110) crystal planes of sieved sepiolite. The orthorhombic structure of sublimed sulfur was confirmed by reference pattern (JCPDS No. 08-0247). On the other hand, the PAni sample possessed an amorphous nature without sharp crystalline peaks. After the impregnation of sulfur, the diffraction peaks of S/SvSp/PAni and S/PTSp/PAni are well matched with bare sublimed sulfur, which indicates the homogeneous distribution of the active material.

### 3.2. FTIR Analysis

**Figure 2:**
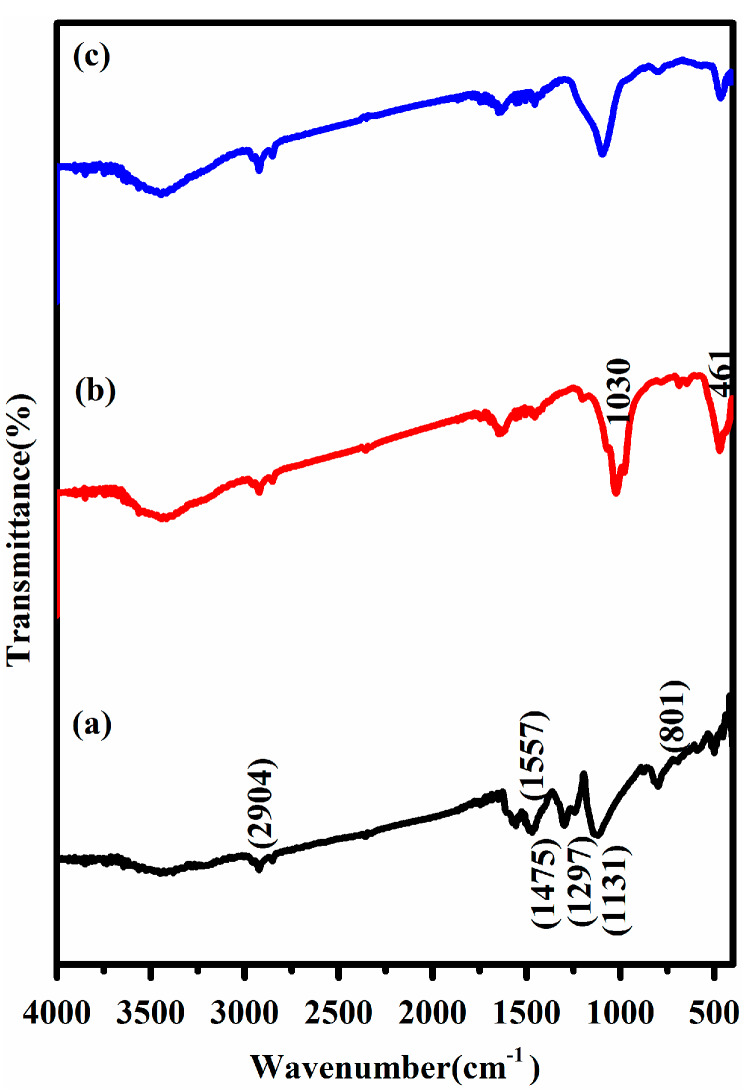
Spectrum of (**a**) PAni (**b**) sulfur/sieved sepiolite/PAni (S/SvSp/PAni) and (**c**) sulfur/post-treated sepiolite/PAni (S/PTSp/PAni).

The FTIR spectra of S/PTSp/PAni composite materials are as shown in Figure 2, which exemplified the occurrence of chemical bonds and functional groups of the composite material in the range of 4000 to 400 cm^−1^. The elemental sulfur was confirmed by the C–S stretching vibration band appeared around 500 cm^−1^. The vibrational peaks appeared in the range 1200–400 cm^−1^ is consigned to the characteristic peak of silicate. The characteristic absorption peak at 1030 cm^−1^ is assigned to Si-O stretching and bending of sepiolite clay mineral [27]. The two peaks arising at 1475 and 1557 cm^−1^ are due to the benzenoid and quinoid-ring vibration of PAni, which are clearly indicating the oxidation state of emeraldine base polyaniline [28]. The infrared peak at 1131 cm^−1^ is a vibrational mode of –N=quinoid=N– stretching and it confirmed the conducting state of polyaniline deposited on the surface of S/PTSp composite [29]. The vibrational peak at 2904 cm^−1^ is attributed to the N–H stretching mode of PAni. Therefore, the FTIR analysis reveals that PAni acts as an excellent connector between active material and porous nature of sepiolite.

### 3.3. Raman Spectroscopy

**Figure 3:**
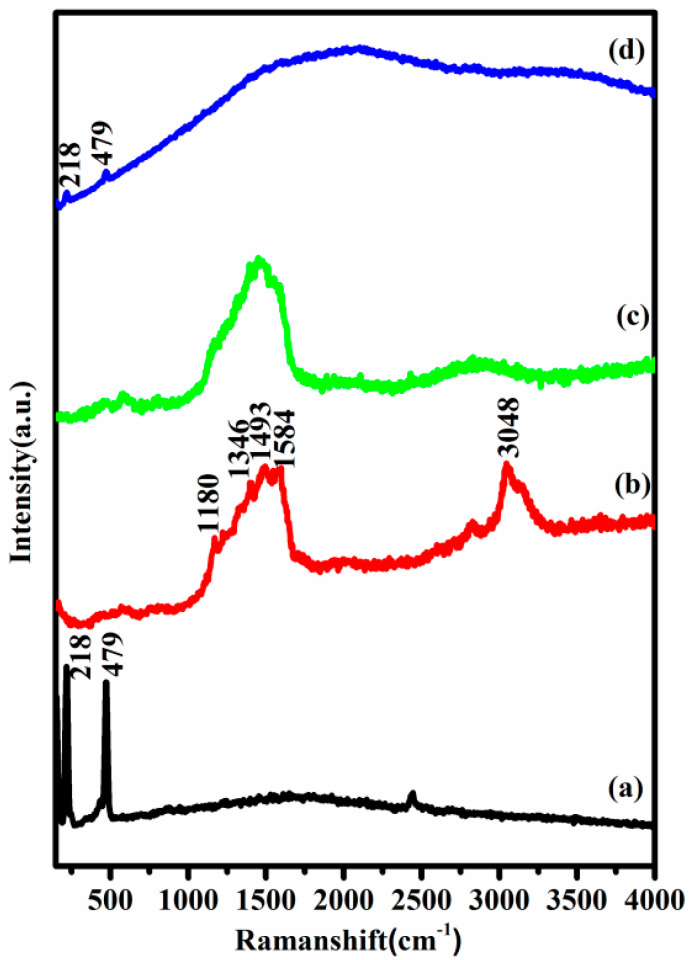
Spectra of (**a**) PS, (**b**) PAni, (**c**) PS/PAni, (**d**) Sulfur/post-treated sepiolite/PAni (S/PTSp/PAni).

Raman spectra of pure sulfur, PAni, PS/PAni and composite are presented in Figure 3. The pure sulfur displays a characteristic peak below 500 cm^−1^ corresponding to A1 symmetry mode of S–S bond. From Figure 3b the quinoid and benzenoid rings are confirmed to the pattern of emeraldine structure of PAni [30]. In Figure 3c, even after the infusion of sulfur into the PAni, the peaks of sulfur are still not visible as it is thoroughly wrapped by PAni. In the S/PTSp/PAni composite, the intensity of the sulfur peaks are very low, signifying the good dispersion of sulfur within the polymer matrix.

### 3.4. Morphological Analysis

#### 3.4.1. SEM Analysis

**Figure 4:**
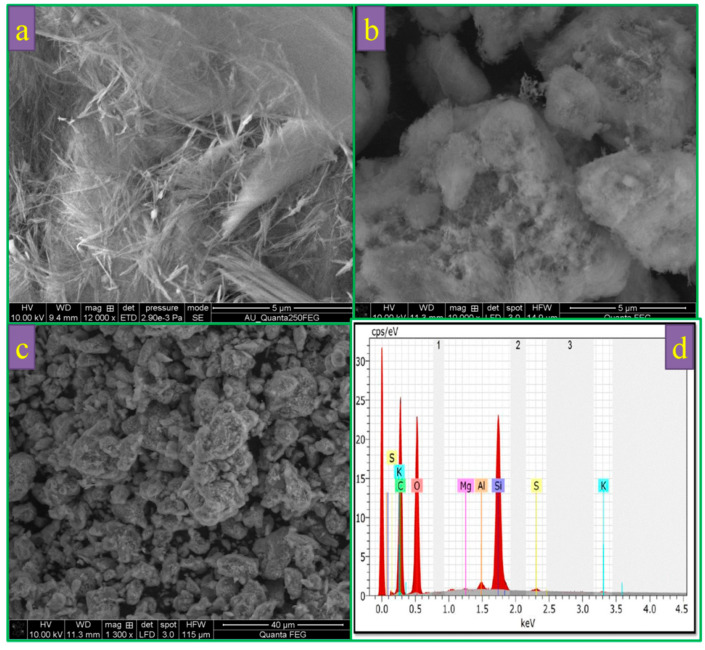
(**a**) Sieved sepiolite, (**b**) sublimed sulfur/ sieved sepiolite/PAni and (**c**) sublimed sulfur /post-treated sepiolite/PAni, (**d**) EDX analysis of composite material (sublimed sulfur /post-treated sepiolite/PAni).

The morphology of sepiolite was further examined by SEM and TEM analyses. From Figure 4a, it is observed that the sepiolite powder consists of short micro-fibrous bundles. Figure 4b,c shows the morphology after sulfur and PAni inserting in sieved and post-treated sepiolite. After sulfur inoculation with closed integration of sulfur and sepiolite was appeared which increases the average diameter of the fibers and also is consistent with the XRD result suggests no structure change in the composite. The presence of sulfur was analyzed by the EDX shown in Figure 4d. Moreover, the element distributions of Si, O, C, and S are in the composite elucidated. The Si element is ascribed to sepiolite, while the element O and C due to polyaniline (PAni). 

#### 3.4.2. TEM Analysis

**Figure 5:**
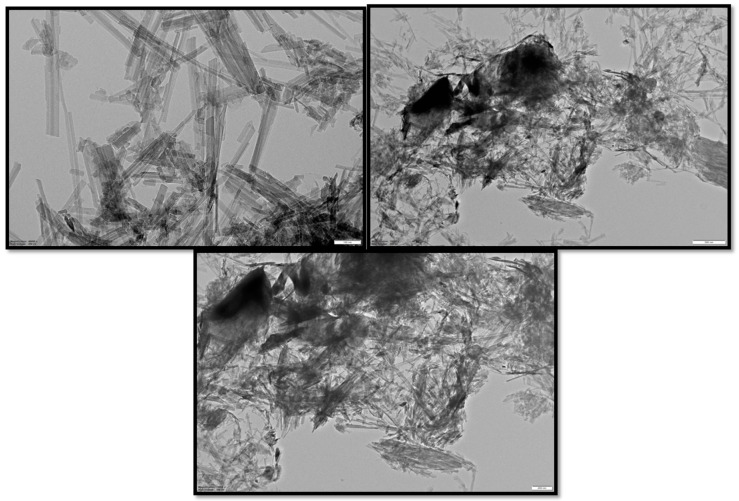
TEM images of composite material with different magnification (sublimed sulfur /post-treated sepiolite/PAni).

The microstructure of the composite cathode found that the sepiolite exhibited needle-like fibrous clusters (Figure 5). After the sulfur interjects, perceptible aggregation appears owing to thermal treatment. The results demonstrate that PAni has been successfully deposited on the composite material. Since the oxidation reactions can only occur at the S/SvSp/PAni and S/PTSep/PAni samples interface during charging/discharging process, the highly uniform distribution of PAni coating can increase the contact area between PAni and the electrolyte, and hence increase the number of active sites for electrochemical reaction in Li–S batteries [31,32].

### 3.5. X-Ray Photoelectron Spectroscopy (XPS)

**Figure 6:**
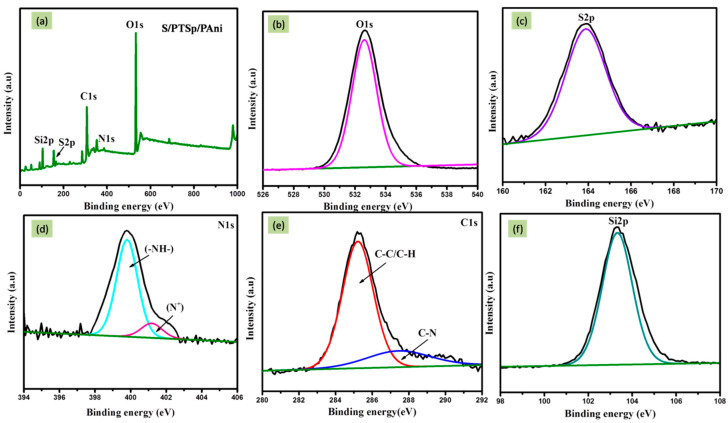
(**a**) Wide spectrum of composite material (sublimed sulfur /post-treated sepiolite/PAni), XPS Spectra of (**b**) oxygen, (**c**) sulfur, (**d**) Nitrogen, (**e**) carbon, (**f**) silicon.

To identify the chemical interaction between the as prepared composite, XPS technique was carried out. The elements of carbon and nitrogen are originated from the backbone of emeraldine PANi. Figure 6a shows the wide spectrum of composite material. Figure 6b–f represents the elemental composition of S/PTSp/PAni composite material revealing the presence of O, N, Cl, S, and Si elements. The peaks are positioned at 532.6, 400.0, 285.4, 164.1, and 103.3eV ascribed to O1s, N1s, Cls, S2p and Si2p respectively. Figure 6c depicts the high resolution XPS spectra of S2p3/2 indicating the valence state of sulfur. The elemental sulfur peak located in the region of 164 eV is assigned to S-S bond [33]. Figure 6d determines the de-convoluted peak of N1s spectra. The Nitrogen contributions are presented at 401.1 and 402.6 eV can be attributed to the oxidized amine and protonated imine respectively. The most important factors of doping and oxidation levels are affecting the electrical and other properties of polyaniline. Figure 6e represents those spectra of silica confirming the existence of sepiolite in the composite material.

### 3.6. Nitrogen Adsorption-Desorption Isotherms

**Figure 7:**
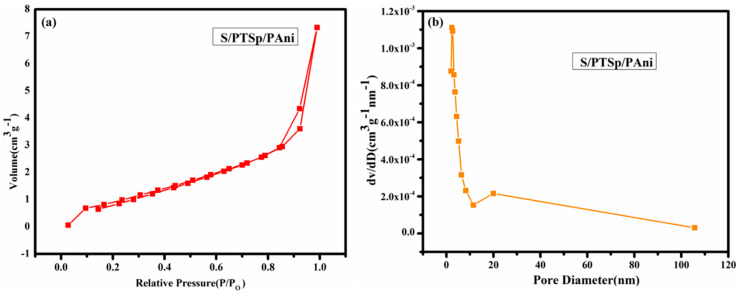
(**a**) Nitrogen sorption isotherms of the composite material (sublimed sulfur /post-treated sepiolite/PAni) (**b**) BJH pore size distribution of (sublimed sulfur /post-treated sepiolite/PAni).

Figure 7 displays the adsorption-desorption isotherms and density functional theory (DFT) pore size distribution plots of obtained cathode material. Porosity of the composite material was further illustrated by BET adsorption isotherm capacity performed at 77K using liquid nitrogen. The BET surface area of the as prepared composite material was calculated 5.282 m^2^/g and the pore volume is 0.012 cc/g. In their terms, the type I isotherm appeared in the prepared material through the International Union of Pure and Applied Chemistry (IUPAC). In DFT method, the pore size can be confirmed at 2.329 nm. The prepared sample exhibits a hysteresis loop obtained at medium relative pressure (p/p0), that indicating the formation of exclusive mesoporous structure. During cycling, the as-prepared mesoporous material allowing the accessibility of electrolyte and favors the rapid transport of lithium ions and also large pore volume indicates that the sulfur has been entrenched in the pores of the composite material.

### 3.7. Electrochemical Studies

**Figure 8:**
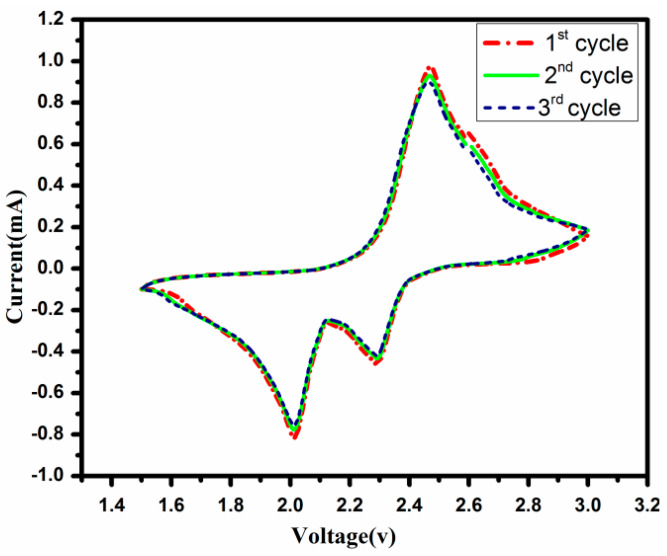
Voltammograms of sublimed sulfur /post-treated sepiolite/PAni) composite in the potential window from 1.5 to 3.0 V (verses Li^+^ /Li).

Figure 8 shows the cyclic voltammogram of S/PTSep/PAni composites of first 40 cycles in the voltage window of 1.5–3.0 V (V vs. Li/Li^+^) at a scanning rate of 0.1 C. In the composite material, the curves show two cathodic peaks (reduction) and one anodic peaks (oxidation) present in the lithium sulfur battery during charge–discharge process. The sulfur loading electrode clearly displays the discharge plateaus of 2.29 and 2.01 V, representing those two-step conversion reactions [34,35,36,37]. The cathodic peak at 2.29 V communicates the transformation of cyclo-octa sulfur (S8) to long chain lithium polysulfides (Li_2_S_X_, 4≤ n ≤8) at the higher potential. The strong reductive peak around 2.01 V suggests further reduction of soluble polysulfide anions appearing as unsolvable short-chain multi lithium sulfides Li_2_S_n_ to Li_2_S/ Li_2_S_2_ at the lower potential [38]. These two constant discharge plateaus were reached at high current density, signifying kinetically proficient charge transfer in the electrode. During the anodic plateau, a broad and overlapped peak is observed at 2.46 V indicating the multi lithium sulfide and Li_2_S have oxidized into elemental sulfur [39,40].The composite material shows no apparent change during the redox peak current and potential plateaus which specify the excellent reactive reversibility and cycling stability. This process can lead to well distributed sulfur in the composite material.

**Figure 9:**
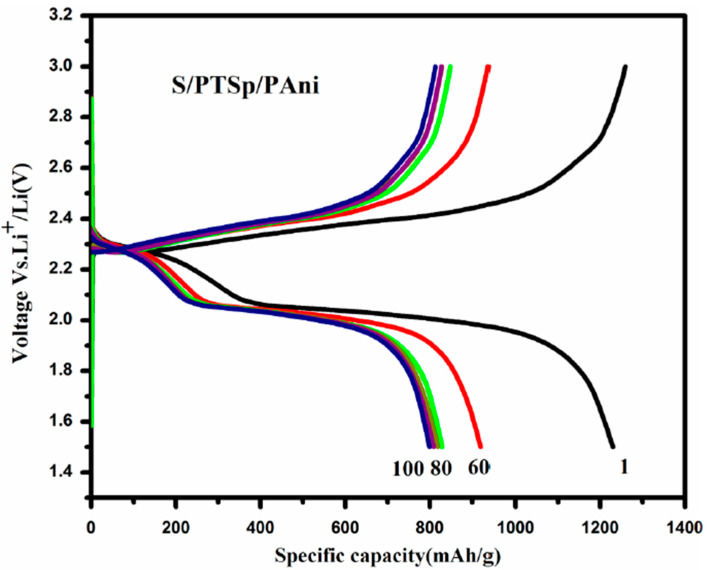
Galvanostatic discharge–charge curves for the S/PTSp /PAni composites at 0.1 C rate.

Figure 9 shows the charge–discharge curves at different cycles for S/PTSp/PAni cathode under 0.1 C rate which is usually in concurrence with the CV results. It is observed that the composite cathode delivers a high specific capacity of 1230 mA h g^−1^ at first cycle and then after 100 cycle the discharge capacity attains 826 mA h g^−1^ for S/ PTSp/ PAni. Conversely, elemental sulfur finely dispersed in this conducting polymer (PAni) and post-treated sepiolite is helpful for cycling stability and high specific capacity. There are two reasons to point out the composite material shows better cyclability over 100 cycles: (i) There may be some dissolution and shuttling of the S species (ii) S/PANi polymer framework shows electrochemically active in situ formation, only when they come into contact with adequate electrolyte so the disulfide bonds divide and recombine during charge/discharge process. It is promising that some of the dissociate disulfide bonds cannot improve to the original cross-linked state, lessening in the strength of the polymer matrix [41]. In the composite material, the post-treated sepiolite has naturally high porosity and a large surface area can enhance dissolved polysulfide sulfur and restrain the volume changes in charge and discharge processes. The capacity retention was calculated from the last discharge cycle by first charge cycle into 100%. After consequent cycles, the discharge capacity of the composite cathode attained capacity retention of about 66%, which is ascribed to the detachment of surface sulfur that dissolved into the electrolyte [42].

**Figure 10:**
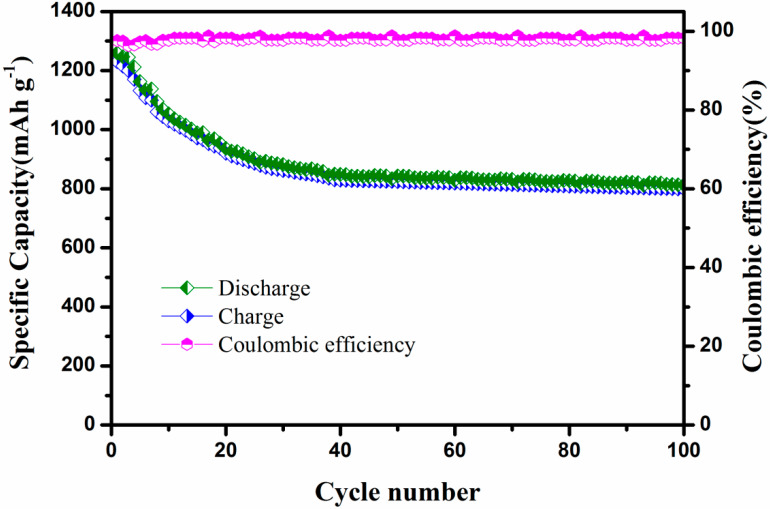
Performance and the responding coulombic efficiency of S/ PTSp/ PAni Composites under discharge rate of 0.1 C.

Figure 10 shows the coulombic efficiency of S/PTSp/PAni cathode at a current density of 0.02 C. The cell exhibited a high and stable coulombic efficiency of 97% at its 40 cycles was obtained from composite material. The insignificant loss of active material due to the lithium polysulfide dissolution prevents shuttling mechanism during discharging processes [43]. This efficiency shows a good reversible electrochemical performance of the cathode material at high sulfur loading, implying that the united effect of volume expansion and porosity of the post-treated sepiolite and chemical functioning of PAni are the most importance factors, and this cathode has high potential to be applied to the battery application [44].

**Figure 11:**
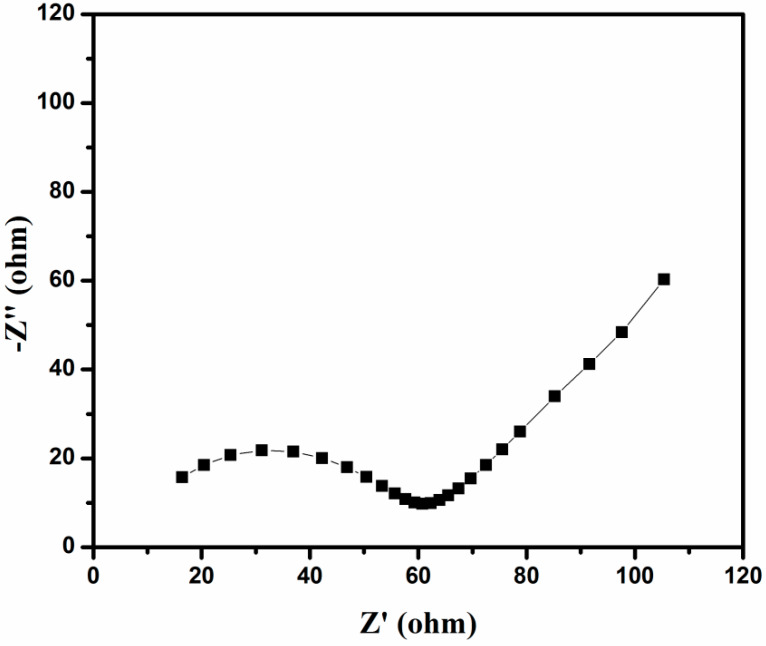
Plot for S/ PTSp/ PAni Composite material.

Figure 11 displays the Nyquist plot of as prepared sample with the frequency ranging from 100 KHz to 0.01 Hz. The semicircle was presented in Nyquist plot at high frequency which is related to the charge transfer resistance (R_ct_). The semi-infinite Warburg diffusion process (Z_w_) indicates that the sloped line in the low frequency region. In long cycling process, the charge transfer resistance of the cathode is slightly varied due to the irreversible deposition and aggregation of insoluble reduction products (Li_2_S_2_ and Li_2_S) on the surface of the composite material, which are not considerable for fully charged state that leads to the high-rate potential of the cathode during long cycling.

## 4. Conclusions

The S/PTSp//PAni and S/SvSp//PAni composite cathode materials have been prepared by the thermal activation method. The TEM results demonstrate that PAni has been successfully deposited on the composite material. The high resolution of XPS spectra depicts the S2p_3/2_ state_,_ indicating that the valence state of sulfur and the nitrogen contributions are attributed to the oxidized amine and protonated imine respectively. Owing to the stable network structure of S/ PTSp/ PAni cathode exhibits an initial specific capacity of 1230 mA h g^−1^ at 0.1 C current density and maintained up to 771 mA h g^−1^ even after 100 cycles with the coulombic efficiency of 97%. Porous structure of sepiolite could be used to hold polysulfides and to diminish the dissolution of polysulfides as well as the loss of active sulfur in cathode. This leads to a substantial improvement in the electrochemical performance of the electrode employed in Li–S batteries. This suggests that PAni could improve the conductivity of the electrode and accommodate the volume change of the sulfur during cycling.
